# Supplementary material for: Causal Relationship between Aging and Anorexia Nervosa: A White-Matter-Microstructure-Mediated Mendelian Randomization Analysis
Source: Biomedicines. 2024 Aug 16;12(8):1874. doi: 10.3390/biomedicines12081874 (PMC11351342; doi:10.3390/biomedicines12081874)
Supplement: Supplementary file 1 [file biomedicines-12-01874-s001.zip › Supplementary Figure/Figure S8/Leaveoneout plot of MR analysis for TL to ubm-b-1498.pdf]

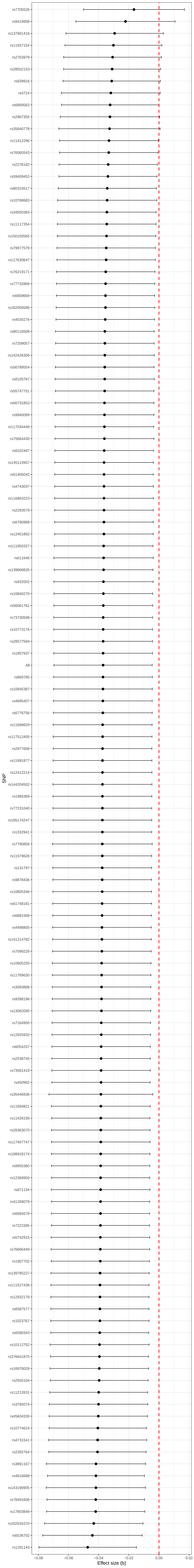
rs7705526
rs9419958
rs137901416
rs11557154
rs2763979
rs28502153
rs939916
rs4724
rs6669563
rs2967355
rs35640778
rs11412296
rs76065543
rs2276182
rs59409453
rs80324517
rs10768683
rs34550383
rs11117354
rs150150565
rs79977579
rs117630647
rs76219171
rs77732866
rs6659669
rs182059586
rs4530278
rs80116508
rs7209057
rs142426306
rs56799554
rs8105767
rs55747751
rs66731853
rs9940099
rs117034449
rs75664430
rs8102497
rs145114957
rs61405042
rs4743037
rs116863223
rs2293579
rs6790988
rs12451892
rs111950327
rs611646
rs139669835
rs932002
rs10840270
rs56061761
rs73730598
rs10773176
rs28577594
rs1957937
All
rs869785
rs10845387
rs4695407
rs6776756
rs11699829
rs117512405
rs2977608
rs11991877
rs12412214
rs144204502
rs1985369
rs77231040
rs185174247
rs1332941
rs77790856
rs11579626
rs131797
rs9878436
rs10805346
rs61748181
rs6881568
rs4498805
rs141214782
rs7099229
rs10905255
rs11769630
rs3093888
rs9398196
rs13062095
rs7164950
rs12925933
rs6054257
rs2538745
rs73581419
rs450962
rs35446936
rs11584821
rs11426156
rs28363070
rs117407747
rs188918174
rs9955360
rs12369950
rs871134
rs41269079
rs6584579
rs7221585
rs5742915
rs76666449
rs1907702
rs139795227
rs111527438
rs12932179
rs6587577
rs1023767
rs6590343
rs10112752
rs376641875
rs16978028
rs2555104
rs11212631
rs3785074
rs45604339
rs10774624
rs4731541
rs2282764
rs3891167
rs4616688
rs143190905
rs78491606
rs17803849
rs202034370
rs6536702
rs1291143
SNP
-0.08
-0.06
-0.04
-0.02
0.00
0.02
Effect size (b)
